# Serial Mediation of Environmental Preference and Place Attachment in the Relationship between Perceived Street Walkability and Mood of the Elderly

**DOI:** 10.3390/ijerph17134620

**Published:** 2020-06-27

**Authors:** Chongxian Chen, Weijing Luo, Ning Kang, Haiwei Li, Xiaohao Yang, Yu Xia

**Affiliations:** 1 Department of Landscape Architecture, College of Forestry and Landscape Architecture, South China Agricultural University, Guangzhou 510642, China; chongxian@scau.edu.cn (C.C.); luoweijing@stu.scau.edu.cn (W.L.); hiwaylee@stu.scau.edu.cn (H.L.); 2 Department of Landscape Architecture, School of Architecture, Tsinghua University, Beijing 10084, China; ningkang@mail.tsinghua.edu.cn; 3 Department of Landscape Architecture, School for Environment and Sustainability, University of Michigan, Ann Arbor, MI 48104, USA; xiaohaoy@umich.edu

**Keywords:** mood, elderly, street environment, place attachment, environmental preference

## Abstract

Urban streets are important public spaces for daily activities that play a crucial role in promoting health in the elderly. The purpose of this study was to investigate the association between perceived street walkability and mood in the elderly, and specifically, the mediating effect of environmental preference and place attachment. We surveyed a total of 269 elderly residents from six streets in Guangzhou, China. We collected assessments of the street environments, environmental preferences, place attachment, and mood status from the elderly. A serial multiple mediator model was constructed using the structural equation modeling method. The results showed that the perceived street walkability was sequentially associated first with an increased level of place attachment (β = 0.798, SE = 0.358, *p* < 0.000) and then environmental preference (β = 0.191, SE = 0.033, *p* = 0.038), which was in turn related to improvement of positive mood in the elderly (β = 0.595 SE = 0.341, *p* < 0.000). Environmental preference alone was found to be significantly associated with positive mood (β = 0.595, SE = 0.341, *p* < 0.000), while no significant effect of place attachment was found when considered individually (β = −0.075, SE = 0.089, *p* = 0.395). These findings provide a greater understanding of the possible mechanism through which street environment impacts mood in the elderly. Therefore, when promoting the emotional experience of the elderly, we might consider not only physical environment factors but also psychological conditions in street environments.

## 1. Introduction

As the aging population rapidly increases, the mental health status of this group has become a pressing issue around the world [1]. It is widely documented that depression and anxiety have become the most common mental health issues among older adults [2,3,4]. Specifically, a 2019 survey on the mental health of older adults in China found that only 30.3% of the elderly who live in urban environments do not have mental health issues [5]. Improving the mental health of the elderly has become an urgent need on the healthy aging agenda. 

Many empirical studies have shown that both objective and perceived built environment are linked to mental health through their impact on physical activity, social connections, and access to green space [6,7,8,9]. In particular, urban streets have been recognized as one of the main public open spaces for daily activities playing a crucial role in promoting health in the elderly [10,11]. However, most previous studies mainly focused on the association between street walkability and the physical health of people [12,13]; only one study has examined the link between objective measured neighborhood walkability and mental health in the elderly such as depression and anxiety in China [14]. 

Furthermore, two main mechanisms have been well-documented through which walkability may confer health outcomes, i.e., through providing a walkable environment for promoting outdoor activities such as walking and for social contact, thereby, improving physical and mental health [15,16,17]. However, there has been little research so far examining the effect of street environment on elders’ mental outcomes including psychological mediators. Although many researchers have found mediating effects of environmental preference and place attachment on the relationship between people and their surrounding landscapes [18,19,20,21], it is still unclear how these psychological factors mediate the pathway through which perceived street walkability impacts on the mood of elderly. Moreover, perceptions of the environment reflect how people interpret their surroundings, which may be influenced by individual factors such as place attachment, local culture, prior experiences, and physical health [22,23]. Therefore, it is likely that older residents form different mental maps of the street environment and behave differently compared with most of the younger population. 

To help address the above research gaps, we have taken the city of Guangzhou as a case study to examine the association between perceived street walkability and the mood of the elderly. We further examined the potential mediating effects of environmental preference and place attachment on the relationship between perceived street walkability and mood. Based on the results, we propose several suggestions for future landscape planning and design of urban streets.

## 2. Literature Review and Theoretical Framework

### 2.1. Perceived Street Walkability and Mood

A large amount of evidence has proved the associations between street walkability and various health outcomes [24,25,26]. For example, effective design of urban streets with adequate seating and smooth pavements additionally makes older people feel safe and encourages their decision to go out, which may subsequently support their independence and help social interactions [10,27]. Conversely, streets with litter and those that are surrounded by high-rise buildings can limit the outdoor activity of older people and significantly impact their quality of life [16,28]. 

During the past decade, most of the existing research has objectively measured street walkability such as land-use mix, trees and vegetation, pavement quality, and intersection density [13], little research has explored the relationship between health and perceived street walkability [29]. Although objective street environment measures play a vital role in guiding future policy and practice, perceived street environment attributes still need to be investigated, as independent associations have been found between the objective and perceived measures with the same street environment attributes [30,31,32]. It means that the impact of perceived street walkability on health may be different from that of the objective street walkability.

Furthermore, a growing body of study has suggested that a built environment is related to traits of psychological well-being such as mood, anxiety, and depression [33,34,35]. For example, Wang et al. found that older people living in a walkable community were more likely to report a positive mood than those who are living in a community with poor walkability [36]. Moreover, a previous study has suggested that perceived neighborhood environmental characteristics could affect the quality of life and mental health [37]. Therefore, it is possible that the way in which older residents assess the walkable environment of a street affects their emotion and attitude toward this street. Based on the existing research, we proposed the following hypothesis:

**Hypothesis** **1.** *Perceived street walkability is significantly associated with mood in the elderly.*

### 2.2. Place Attachment

Place attachment is a concept in the field of environmental psychology that has been described as the strong emotional bond between a person and a particular place or environment [38,39]. Previous visiting experiences and memories are important factors that promote the cultivation of an individual’s place attachment to a place or setting [40]. Although many definitions of place attachment have been proposed, the most commonly used construct involves two underlying factors of place identity and place dependence [21,41,42]. Place identity refers to one’s relationship with the physical environment or place as an extension of the self [43]. These dimensions of the relationship are defined by the complex emotional bonding to the environment through the individual’s ideas, beliefs, feelings, values, and behavioral tendencies [42]. In contrast, place dependence is a functional attachment that reflects a particular setting to satisfy a user’s goal and desired activity needs [44]; an assessment of how well this setting is able to satisfy these needs [45]. Researchers have suggested that place identity and place dependence are parallel dimensions of place attachment and should both be included in the measurement of place attachment [42,46]. 

In previous studies, place attachment was found to be linked with the characteristics of a landscape. For instance, some researchers found that people demonstrated greater place attachment when they experienced a landscape with strong local characteristics or landscape elements [47,48]. This effect has also been found in studies showing that immigrants prefer to name places and design houses’ environmental characteristics in a way that closely represents their cultural identity [49]. These findings provide evidence for the significance of environmental characteristics and qualities in building place attachments. 

Furthermore, many studies have shown that enhanced attachment to certain places can improve mental health in people [50,51], further justifying the significance of place attachment to mental health in both natural and urban environments. One study found greater self-reported attachment to local green space and improved mental health of residents in the city of Groningen [52], whereas another study found that exposure to nature improved cognitive capacity when nature was consistent with a salient identity [53]. Other research has suggested that place attachment plays a positive role in both nature and the urban environment for restorative perceptions [50], while attachment disruption may lead to negative feelings of grief or loss [54]. Moreover, a few studies have shown the mediating role of place attachment on the relationship between people and their surrounding environments [55,56,57,58]. For example, Isa et al. and other scholars have found the mediating role of place attachment on the relationship between environment satisfaction and revisit intention [57,58]. Other studies also suggested that place attachment partially mediates the links between the natural environment and mental health [59,60]. We therefore propose a second hypothesis:

**Hypothesis** **2.** *Place attachment is a potential mediating variable between perceived street walkability and the mood of the elderly.*

### 2.3. Environmental Preference

In environmental psychology, environmental preference is an affective tendency toward either favorable or attractive environments [61,62,63]. Previous research has defined environmental preference as being reflective of perceptual mechanisms that allow the individual to assess the environment in a rapid and automatic manner [18]. Kaplans’ information processing theory argued that humans prefer environments containing information that will make them understand their surroundings effectively and enjoyably, which is crucial for human survival from an evolutionary perspective [61]. The environmental preference model postulated that preference helps to satisfy two basic human needs—to understand and to explore, which also regarded as two affectively informational outcomes. Kaplan explained environmental preference by using a matrix containing two concepts, which were labeled as “Understanding” (to comprehending the settings, indicating the settings should provide a feeling of coherence and legibility) and “Exploration” (being attracted by the settings due to complexity and mystery) [61]. Moreover, coherence, legibility, complexity, and mystery were proposed as predictors of environmental preference [61], which can predict aesthetic appraisal and reflect preference as measured by the beauty [64]. It is likely that landscape characteristics may induce people’s preference for a place through stimulation of coherence, legibility, complexity, and mystery. 

Therefore, environmental preference is not only associated with physical landscape characteristics and environmental attractiveness but also with the subjective assessment of their features [65,66,67,68]. For instance, Aspinall et al. explored the environmental attributes relevant to older people’s preference for a neighborhood park and found that environmental attributes in line with their preference were lack of nuisance, presence of facilities, and the presence of trees [68]. Some studies also suggested that environmental preference is closely related to mental health and well-being, indicating that meeting environmental preferences can promote the restorative potential of the environment [19,69,70,71]. It was also found that preferences can mediate the relationship between scenic beauty and restoration [72,73]. Given these results, we proposed the third hypothesis in our study:

**Hypothesis** **3.** *Environmental preference is a potential mediating variable between perceived street walkability and the mood of the elderly.*

Furthermore, some researchers have argued that place attachment is associated with environmental preference [74,75]. Indeed, some relevant studies suggest that affective attachment to the setting has an influence on individuals’ environmental preference [76,77], which is defined as “a representation of place identity” [69]. It means that individuals usually prefer environments with which they are familiar [78]. In support of this, research has demonstrated that the mediating role of environmental preference and place attachment in the relationship between the natural environment and restorative outcomes [60]. We therefore propose a final hypothesis:

**Hypothesis** **4.** *The relationship between perceived street walkability and the mood of the elderly is mediated simultaneously by both place attachment and environmental preference.*

**Figure 1:**
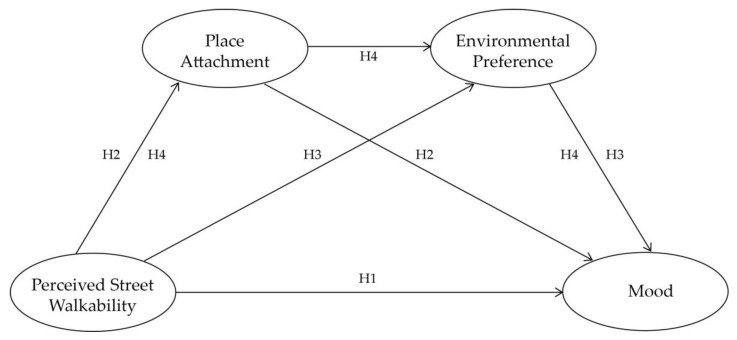
Theoretical framework of the relationship between perceived street walkability and the mood of the elderly, with place attachment and environmental preference as mediators.

Based on the literature review and hypotheses presented above, we proposed the theoretical framework in Figure 1, depicting how the perceived street walkability affects the mood of the elderly directly and indirectly through a specific mediating working pathway, which uses both place attachment and environmental preference as mediators.

## 3. Materials and Methods 

### 3.1. Study Design and Participants

Guangzhou is one of the highest-density cities in south China and includes a total of 136 subdistricts across which the demographic and socioeconomic profiles vary widely. According to the investigation “2018 Report on the Development of Aging Agenda in Guangzhou”, the population proportion of individuals over 60 years of age is more than 20% [79]. 

**Figure 2:**
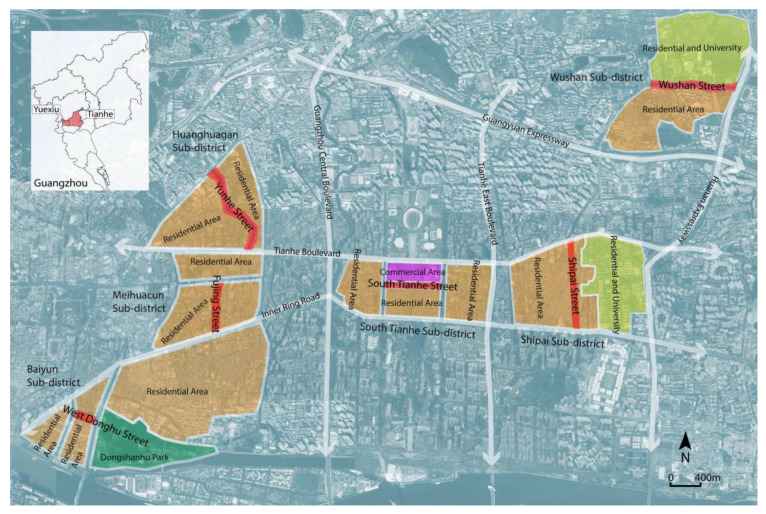
Location of six streets in Guangzhou.

**Figure 3:**
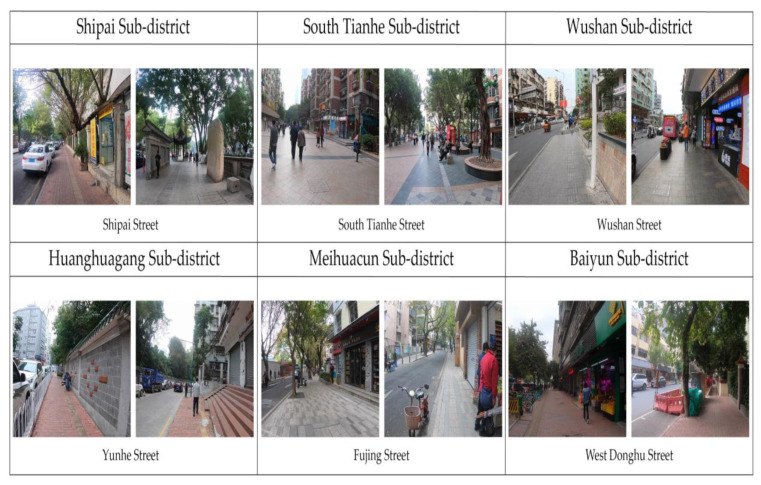
Photographs of six different street environments.

Study areas were preliminarily selected from Tianhe and Yuexiu districts because residential neighborhoods in these areas have a relatively high proportion of the older population [80,81]. Then, a field trip was conducted in these areas’ streets to investigate the activity status of the older population and narrow down the specific study sites. Finally, six streets from the six subdistricts in Guangzhou were selected as the typical cases for this study (Figure 2 and Figure 3). Using a convenience sampling strategy, we approached older people on the streets, informed them about the objectives of the study, and asked about their age and willingness to take the survey. Residents who were aged 60 years or above and able to understand the survey questionnaire were invited to complete an on-site questionnaire survey. The anonymous questionnaire was administered to the participants by trained researchers. Before data collection, consent was obtained from each participant. We conducted a face-to-face questionnaire survey during the period of 3 December 2019–20 December 2019. A total of 305 residents were invited to participate and 275 on-site questionnaires were filled out, indicating a 90.17% response rate, and 30 participants (9.83%) refused to cooperate during the survey. Among the collected questionnaires, six respondents were excluded from this study because of incomplete responses, resulting in a total of 269 valid questionnaires.

### 3.2. Measurements

#### 3.2.1. Perceived Street Walkability

Based on the Neighborhood Environment Walkability Scale (NEWS) and elderly friendly urban street evaluation in China [82,83], the perceived street walkability was assessed using a five-point Likert scale that comprised five categories: 1) land use mix-access (three items); “easy to walk to public transport (L1)”, “easy to walk to public facilities (L2)”, and “easy to walk to recreational facilities (L3)”; 2) street connectivity (two items); “walkways connecting to street (S1)” and “short distance between intersections (S2)”; 3)infrastructure for walking (three items); “pavement in good condition (I1)”, “footpath greening isolation from the traffic (I2)”, and “footpath is easily accessible (I3)”; 4) aesthetics (five items); “greening in good quality along footpath (A1)”, “pavement clean and free from litter (A2)”, “human-scale street space (A3)”, and “pleasant environmental features along footpath (A4)”, “buildings in good quality along footpath (A5)”; and 5) safety (two items); “appropriately well-lit at night (F1)” and “slow traffic on nearby street (F2)”. Items were scaled from 1–5, with higher scores indicating higher favorability.

#### 3.2.2. Positive and Negative Mood

Mood was assessed using the Positive and Negative Affect Schedule (PANAS), which comprises two subscales to measure positive and negative mood [84,85]. Measurement data in previous studies showed considerable construct validity and reliability of the scale [86]. The 18-items version of PANAS has shown high reliability for Chinese use [87] and was administered in the current study. This scale presents nine positive-affect items (PA; e.g., joyful) and nine negative-affect items (NA; e.g., nervous). A five-point Likert scale asks participants to rate their mood status over the past two weeks.

#### 3.2.3. Environmental Preference

Preference of the street environment of the elderly was assessed based on the environmental preference matrix proposed by Kaplan [88]. The predictor variables of the environmental preference matrix were defined by the two major categories of understanding and exploration in the perceptual process [64]. The environmental preference matrix comprises four variables: (1) coherence: “The components of the street environment seem to hang together (C1)”, ”The street environment has repeated elements (C2)”, and “The component of the street environment help each other to organize a well-arranged scene (C3)”; (2) legibility: “It is easy to figure out the scene of the street environment (LL1)”, “It is not easy to get lost in the street environment (LL2)”, and “The street environment has distinct markers (LL3)”; (3) complexity: “The street environment has too many intricate elements (CC1)”, “The street environment contains abundant elements and features (CC2)”, “The street environment lacks rule and order (CC3)”, and “The scene of the street environment is changeful (CC4)”; and 4) mystery: “The street environment makes me feel there is an interesting scene to explore (M1)”, “The scene of the street environment is circuitous (M2)”, “The scene of the street environment is far-reaching and mysterious (M3)”, and “The scene of the street environment makes me want to navigate more (M4)”. We applied the instrument using four predictor variables that were presented in the modified Chinese version of the scale [89]. Participants indicated their responses using a five-point Likert scale ranging from strongly disagree to strongly agree.

#### 3.2.4. Place Attachment

The Place Attachment Scale (PAS) developed by Williams and Vaske [38] was used to measure the level of place attachment of the elderly to their surrounding street environments. The scale is designed to evaluate the extent of one’s emotions and feelings for a place. In this study, a revised version of the 10-item PAS with Chinese content was used [90] and included dimensions of place dependence: “The street is the best place for what I like to do (PD1)”, “No other place can compare to this street (PD2)”, “I get more satisfaction out of visiting this street than any other (PD3)”, “I wouldn’t substitute any other street for doing the types of things I do here (PD4)”, and “Doing what I do here is more important to me than doing it in any other place (PD5)”, and place identity: “I feel this street is a part of me (PI1)”, “This street is very special to me (PI2)”, “I identify strongly with this street (PI3)”, “I am very attached to this street (PI4)”, and “Visiting this street says a lot about who I am (PI5)”. Participants indicated their responses using a five-point Likert scale ranging from strongly disagree to strongly agree.

### 3.3. Statistical Analyses

Data were analyzed using SPSS software (SPSS 25.0 version, IBM, Armonk, NY, USA) and Amos 21 software (IBM, Armonk, NY, USA). We analyzed descriptive statistics for both demographic and experimental variables. We tested the reliability and validity of the construct by examining the Cronbach’s α coefficients and performing a confirmatory factor analysis (CFA). Composite reliability (CR) coefficients, average variance extracted (AVE) scores, and AVE square roots were calculated as estimates. Spearman’s correlation analysis was performed to examine the general relationships among the five variables—street environment, environmental preference, place attachment, positive mood, and negative mood. A structural equation model (SEM) was built to examine the theoretically indicated hypotheses, using a maximum-likelihood (ML) estimator. The bias-corrected percentile method was used to examine the total, direct, and indirect effects in the model. We conducted bootstrap analyses with 95% confidence intervals (95% CI) at 5000 samples. If the 95% confidence interval did not contain zero, the indirect effect was considered significant, indicating that the proposed mediating variable mediated the effect of the independent variable on the dependent variable.

## 4. Results

### 4.1. Demographic Characteristics

The sociodemographic characteristics of 269 participants are presented in Table 1. Over half of the participants were female (53.5%). Most of the participants were aged above 70 years (65.4%), followed by 20.4% of the individuals who were aged between 65 and 69 years. Of note, 45.0% of participants worked in the public sector before retirement. In terms of educational status, the participants covered various education levels, and most of them (83.3%) were high school graduates or below that.

### 4.2. Preliminary Analyses

Table 2 shows the standardized path coefficients, Cronbach’s α values, CR coefficients, and AVE scores of each variable under analysis. Standardized path coefficients, such as correlation coefficients, were used to assess the relationship between variables on an abstract scale [91]. The analyses showed that the standardized path coefficients of each indicator ranged from 0.189 to 1.012. Additionally, all of the Cronbach’s α coefficients were regarded as reliable, with values above the threshold of 0.7. Moreover, the CR coefficients for each variable exceeded the threshold value of 0.7 [92], and AVE scores were calculated with values higher than 0.5 [93], indicating that the results were acceptable with convergent validity. The results also showed that the AVE square roots (Table 3) for each variable were greater than those for all the variable correlations, indicating that the study possessed discriminant validity. To sum up, the results indicated that all the variables were well-designed, with high reliability and validity.

Table 3 reports the descriptive statistics and Spearman’s correlations, as well as the discriminant validity between the variables. Participants reported higher positive mood (M = 3.00, SD = 1.12) compared to the negative mood (M = 1.34, SD = 0.47). Moreover, participants tended to have positive assessments of the perceived street walkability (M = 3.33, SD = 0.65). The mean scores of environmental preference and place attachment were 2.34 ± 0.61 and 1.90 ± 0.73, respectively. The correlation analyses indicated that perceived street walkability had a significant positive association with positive mood and was negatively related to negative mood. Moreover, positive relationships were found between environmental preference and place attachment and both were correlated with perceived street walkability and positive mood. The findings of the correlation analyses suggest that some mediating effects may exist between the above variables. However, neither environment preference nor place attachment showed a significant correlation with negative mood, making it unlikely that there is a mediating effect of environment assessment on negative mood; as a result, we only included positive mood in our subsequent mediation models.

### 4.3. Mediation Analyses

The structural equation model provided a satisfactory goodness of fit to the sample data. As shown in Table 4, the values of chi-square/df were small and satisfied the ideal critical criterion. However, some researchers have suggested that the chi-square test is sensitive to sample size, which may produce bias in model fit [94]. Thus, we also used other alternative indexes to assess the model fit. The values of the goodness-of-fit index (GFI) and adjusted goodness-of-fit index (AGFI) were 0.836 and 0.750, respectively, which were marginally lower than 0.9 but above the acceptable value of 0.7, indicating acceptable model fit [94,95]. The value of the root mean square error of approximation (RMSEA) was 0.053, which was lower than the cutoff value of 0.08, suggesting that the model exhibited a good fit to the data [94]. The values of the comparative fit index (CFI) and Tucker–Lewis index (TLI) were 0.945 and 0.920, respectively, which also indicated adequate fit to the data [94].

Table 5 illustrates the results of the path analysis of the latent variables and reports the standardized coefficients, their standard errors, and *p* values. Positive associations were found among most of the variables in this specific mediating pathway. Specifically, the effects of the perceived street walkability on positive mood were significant (β = 0.245, SE = 0.326, *p* = 0.039), indicating that perceived street walkability may be a protective factor for the positive mood of older adults. Perceived street walkability was positively associated with place attachment (β = 0.798, SE = 0.358, *p* < 0.000) and environmental preference (β = 0.644, SE = 0.156, *p* < 0.000), suggesting that the respondents who had positive evaluations of the street walkability may express greater place attachment and preference for the place. Similarly, place attachment had a positive impact on the environmental preference (β = 0.191, SE = 0.033, *p* = 0.038). In addition, the association between environmental preference and positive mood was positive (β = 0.595, SE = 0.341, *p* < 0.000), while no direct association was observed between place attachment and positive mood (β = −0.075, SE = 0.089, *p* = 0.395).

**Figure 4:**
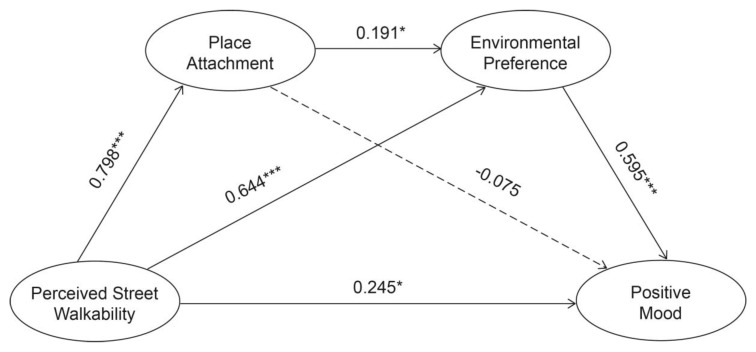
Standardized coefficients in the structural equation model. Note: * *p* < 0.05; *** *p* < 0.001.

As shown in Figure 4, perceived street walkability was significantly associated with place attachment, which was in turn correlated with greater environmental preference and eventually contributed to higher levels of positive mood, indicating that there was a significant serial mediation effect. Additionally, there was a sole mediation effect of the environmental preference on the relationship between perceived street walkability and positive mood. The results generally supported our four hypotheses, with the exception of Hypothesis 2, i.e., that place attachment alone mediates on the relationship between perceived street walkability and the mood of the elderly. 

Table 6 presents the results of the mediating effect of place attachment and environmental preference in the relationship between street walkability and positive mood. The 95% confidence intervals of the total effect, direct effect, and indirect effect did not contain zero. The results indicated that a partial mediation effect was found between street walkability and positive mood in this specific mediating pathway.

## 5. Discussion

In this study, we sought to examine the serial mediating roles of environmental preference and place attachment in the association between perceived street walkability and mood in older adults. First, we found that perceived street walkability showed a strong correlation with mood, particularly for positive mood. Second, we observed that the path through serial multiple mediations of place attachment and environmental preference, as well as the sole mediating role of environmental preference, was statistically significant. The implications of these findings provide a greater understanding of a mechanism that may explain how the street environment impacts mood in the elderly.

Correlation analyses showed that the perceived street walkability quality was significantly correlated with positive and negative mood, which was consistent with previous studies demonstrating that the perceived built environment can trigger mood changes by altering psychosocial processes [85,96,97,98]. However, our study also found that the absolute values of the correlation coefficients of positive mood were generally higher than those of negative mood, indicating that perceived street walkability may be more relevant to positive mood than to negative mood among older people. Furthermore, we found the direct positive effects of perceived street walkability on positive moods. Consistent with this, previous research has shown that viewing a good-quality street environment containing pleasant elements (i.e., trees, light, clean pavement, and street furniture) could promote a positive mood and enhance a sense of pleasure [17,99,100,101]. On the other hand, the perception of complexity and poor material conditions in the outdoor environment may have side effects that diminish positive mood [102,103]. 

In addition, the more significant findings of our study were the observation that perceived street walkability had not only a direct effect but also an indirect effect on positive mood via the mediation of place attachment and environmental preferences. Specifically, environmental preference as the single mediating variable significantly mediated the relationship between perceived street walkability and positive mood. Based on studies by Kaplan, the cognitive contents of landscape and preference are associated with affective response [104]. One possible interpretation of these findings might be that a favorable setting could fulfill needs in the interactions between older adults and street environment, facilitating the perception of independence and, thus, eliciting greater improvement in the mood [65,105,106]. Surprisingly, the mediation effect of place attachment alone was not significant, suggesting that this pathway remained unclear in this research. This outcome was inconsistent with previous studies, which found that greater place attachment may contribute to a higher level of subjective well-being and positive affective outcomes [39,107]. Furthermore, the respondents in our study showed a relatively low place attachment to the street environment in Guangzhou. The results regarding place attachment were difficult to explain, but it might be partly related to the following possible reasons. It has been reported that Guangzhou has seen an increasing number of urban migrants in recent years [108], and many elderly migrants come to the city with their children to take care of the third generation. The sense of place of elderly migrants may be compromised by their attachment to their hometown and low level of place identity [109,110]. We speculate that the migration and cultural background may counteract the positive effect of one’s place attachment on emotional response, an implication that merits further research.

This specific serial multiple mediation model may explain the potential mechanism between perceived street walkability and positive mood. The results indicated that perceived street walkability was sequentially associated with increased place attachment first and then environmental preference, which in turn contributed to a positive mood in this specific mediating pathway. These findings are in line with previous studies that showed that the perception of a higher-quality level of neighborhood environment has a positive impact on place attachment [111,112,113]. Moreover, many studies have found that place attachment has a positive effect on the attractiveness of the environment [114,115], and individuals with a stronger attachment to their community may show a higher preference for surrounding street environments. In addition to satisfaction with their street environment, social connection and support were also closely linked to the residents’ sense of belonging to the neighborhood, which in turn may have promoted their preference for the place and enhanced their positive emotional experience [18,116].

### 5.1. Implications for Practice

The major contribution of our study is the finding that perceived street walkability could both directly and indirectly affect mood via preference and attachment to the environment, suggesting that the perception of the physical environment and social-psychological conditions of the street environment are possible not independent of each other. The current findings have practical implications in building age-friendly neighborhood environments, which may consequently benefit mental health in the elderly. On one hand, our findings indicated that the perceived street walkability was significantly associated with the mood of the elderly. Thus, city planners might need to consider the importance of the physical conditions of street environments, which may induce a better perception of the street environment in the elderly. For example, landscape greenery, appropriate street furniture, convenient sidewalks, and comprehensive information systems could be provided to create easy access and a friendly street environment. On the other hand, given the mediating role of environmental preference and place attachment in this specific pathway, merely focusing on the improvement of the physical conditions of perceived street walkability maybe not be sufficient for promoting positive emotional experiences. The findings also provide some clues that community administrators might need to pay more attention to the construction of the psychological environment in the urban street. Because residents’ experience in a specific street may vary due to the different intension of their place attachment to the neighborhood. Therefore, policymakers and practitioners could further take measures to foster the elderly’s community identity and strengthen their affinity toward their neighborhoods. For instance, the local community could provide some programs to enhance social bonding among the residents, which may in turn promote people’s place attachment. Finally, some common preferences for environmental features among the elderly might also be considered, such as issues regarding street connectivity, air quality, and safety [65].

### 5.2. Limitations and Future Research

The current study has several limitations that should be acknowledged. First, the cross-sectional nature of this study hinders the definitive establishment of causality among the assessed variables, and future work should incorporate longitudinal or experimental studies to establish casual conclusions that more closely capture the dynamic nature of affective changes and related factors. Moreover, mediation models of cross-sectional data may produce biased estimates of the mediating variables [117,118]. Thus, in the future study, we should use the experimental manipulation of mediators to overcome unobserved variables’ limitation. Second, the study was conducted using a convenience sampling method in Guangzhou and this may limit the generalizability of our study results. The sample could be expanded in the future by including elderly groups from different districts in the city and adopt random sampling to avoid potential bias. Third, the elderly were observed as a collective in this study and distinctions were not examined among some demographic variables (e.g., age ranges, health condition, and registered residence). It is a great challenge for future studies to reveal the similarities and differences in emotional responses related to the neighborhood environment of these specific subgroups. Fourth, we only collected self-reported measures and assessed the perceived street walkability primarily by material aspects of the neighborhood environment. In future research, it would be wise to use a multimodal approach (e.g., interviews and observations) to overcome the common problem of questionnaire surveys and develop a more comprehensive instrument with additional descriptors of functional and social dimensions of the perceived street walkability. Additionally, future research should be undertaken by using objective environmental indicators. Finally, our research only tested the specific mediating working pathway mentioned above, which may provide a limited potential mechanism among the variables. Other pathways could be examined in future work, for example, using perceived street walkability as a mediator in the relationship between place attachment and mood.

## 6. Conclusions

In this study, we investigated the relationship between perceived street walkability and mood among the elderly using a serial multiple mediation approach. Our findings are not only consistent with prior research, but they also shed light on a possible mechanism of the relationship between place attachment and environmental preference. Specifically, our results showed that perceived street walkability was sequentially associated first with a higher level of place attachment and then environmental preference, which was in turn related to increased positive mood. Moreover, we found that place attachment appears to be relatively less important than environmental preference in the mechanism underlying this relationship between environment and mood. Our results provide the direction for future studies toward understanding the relationship between street environment and mental health of the elderly, which may provide a source of reference for planners, designers, and relevant policymakers in the local government to create an age-friendly urban environment.

## Figures and Tables

**Table 1:** Demographic characteristics of the participants (N = 269).

Characteristic	Category	*n* (%)
Sex	Male	125 (46.5%)
	Female	144 (53.5%)
Age (years)	60–64	38 (14.1%)
	65–69	55 (20.4%)
	70–79	108 (40.1%)
	≥80	68 (25.3%)
Occupation (before retirement)	Public institution personnel	121(45.0%)
	Scientific research personnel	6 (2.2%)
	Business and service personnel	32 (11.9%)
	Agricultural production personnel	45 (16.7%)
	Others	65 (24.2%)
Education	High school or below	224 (83.3%)
	Bachelor’s degree	41 (15.2%)
	Master’s degree	2 (0.7%)
	Doctorate	2 (0.7%)

**Table 2:** Reliability and validity analysis.

Variable	Items	Standardized Path Coefficients	Cronbach’s α	CR	AVE
Perceived street walkability	L1 Easy to walk to public transport	0.802	0.879	0.937	0.504
	L2 Easy to walk to public facilities	0.829			
	L3 Easy to walk to recreational facilities	0.607			
	S1: walkways connecting to street	0.945			
	S2: short distance between intersections	0.616			
	I1: pavement in good quality	0.736			
	I2: footpath greening isolation from the traffic	0.714			
	I3: footpath is easily accessible	0.744			
	A1: greening in good quality along footpath	0.702			
	A2: pavement cleanliness and free from litter	0.685			
	A3: human scale street space	0.637			
	A4: pleasant environment features along footpath	0.596			
	A5: building in good quality along footpath	0.740			
	F1: appropriate well-lit at night	0.686			
	F2: slow traffic nearby street	0.489			
Positive mood	PA1 Active	0.909	0.969	0.970	0.786
	PA2 Enthusiastic	0.925			
	PA3 Cheerful	0.896			
	PA4 Joyful	0.957			
	PA5 Excited	0.943			
	PA6 Proud	0.861			
	PA7 Inspired	0.914			
	PA8 Strong	0.808			
	PA9 Grateful	0.743			
Negative mood	NA1 Guilty	0.357	0.845	0.835	0.396
	NA2 Upset	0.410			
	NA3 Scared	0.849			
	NA4 Nervous	0.868			
	NA5 Afraid	0.847			
	NA6 Ashamed	0.189			
	NA7 Irritable	0.478			
	NA8 Jittery	0.773			
	NA9 Angry	0.465			
Environmental preference	C1Each component of the street environment seems to hang together	1.012	0.866	0.940	0.540
	C2 The street environment has repeated elements	0.632			
	C3 Each component of the street environment helps each other to organize a well-arranged scene	0.413			
	LL1 It is clear to figure out the scene of the street environment	0.786			
	LL2 It does not easy to get lost in the street environment	0.866			
	LL3 The street environment has distinct markers	0.781			
	CC1 The street environment has too many intricate elements	0.592			
	CC2 The street environment contains abundant elements and features	0.540			
	CC3 The street environment lacks of rule and order	0.607			
	CC4 The scene of the street environment is changeful	0.909			
	M1 The scene of the street environment make me wonder to navigate more	0.650			
	M2 The scene of the street environment is circuitous	0.849			
	M3 The scene of the street environment is far-reaching and mysterious	0.711			
	M4 The street environment makes me feel there is an interesting scene to explore	0.712			
Place attachment	PD1 The street is the best place for what I like to do	0.767	0.943	0.958	0.696
	PD2 No other place can compare to this street	0.887			
	PD3 I get more satisfaction out of visiting this street than any other	0.889			
	PD4 I wouldn’t substitute any other street for doing the type of things I do here	0.818			
	PD5 Doing what I do here is more is more important to me than doing it in any other place	0.849			
	PI1 I feel this street is a part of me	0.864			
	PI2 This street is very special to me	0.842			
	PI3 I identify strongly with this street	0.693			
	PI4 I am very attached to this street	0.881			
	PI5 Visiting this street says a lot about who I am	0.835			

Note: CR—composite reliability; AVE—average variance extracted.

**Table 3:** Descriptive statistics, correlations, and discriminant validity among the variables.

Variable	Mean	SD	1	2	3	4	5
1. Perceived street walkabilit	3.33	0.65	0.684				
2. Environmental preference	2.34	0.61	0.56 **	0.735			
3. Place attachment	1.90	0.73	0.65 **	0.57 **	0.834		
4. Positive mood	3.00	1.12	0.63 **	0.62 **	0.48 **	0.887	
5. Negative mood	1.34	0.47	–0.19 **	–0.06	–0.07	–0.26 **	0.629

Note: **—Coefficient is significant at the 0.01 level (two-tailed). AVE square roots are bolded on the diagonal. SD—standard deviation.

**Table 4:** Model fit indexes.

Model Fit Index	Ideal Critical Criterion	Acceptable Value	Model Fit Statistics
Chi-square/df	1–2	1–3	1.767
GFI	>0.9	>0.7	0.836
AGFI	>0.9	>0.7	0.750
RMSEA	<0.08	<0.09	0.053
CFI	>0.9	>0.7	0.945
TLI	>0.9	>0.7	0.920

Note: GFI—goodnes-of-fit index; AGFI—adjusted goodness-of-fit index; RMSEA—root mean square error of approximation; CFI—comparative fit index; TLI—Tucker–Lewis index.

**Table 5:** Path analysis.

Path	Standardized Coefficient	SE	*p*
Perceived Street Walkability→Positive Mood	0.245	0.326	0.039
Perceived Street Walkability→Place Attachment	0.798	0.358	0.000
Perceived Street Walkability→Environmental Preference	0.644	0.156	0.000
Place Attachment→Environmental Preference	0.191	0.033	0.038
Place Attachment→Positive Mood	−0.075	0.089	0.395
Environmental Preference→Positive Mood	0.595	0.341	0.000

Note: SE—standard error.

**Table 6:** Model effect indexes.

Variable	Effect	Point Estimate	Bootstrapping 95% CI
Perceived Street Walkability→Positive Mood	Total effect	0.659	(0.583, 0.731)
	Direct effect	0.245	(0.175, 0.496)
	Indirect effect	0.414	(0.200, 0.725)

Note: 95% CI—confidence intervals.
